# Life History Changes in Coral Fluorescence and the Effects of Light Intensity on Larval Physiology and Settlement in *Seriatopora hystrix*

**DOI:** 10.1371/journal.pone.0059476

**Published:** 2013-03-27

**Authors:** Melissa S. Roth, Tung-Yung Fan, Dimitri D. Deheyn

**Affiliations:** 1 Scripps Institution of Oceanography, University of California San Diego, La Jolla, California, United States of America; 2 National Museum of Marine Biology and Aquarium, Pingtung, Taiwan, Republic of China; 3 Institute of Marine Biodiversity and Evolution, National Dong Hwa University, Pingtung, Taiwan, Republic of China; Leibniz Center for Tropical Marine Ecology, Germany

## Abstract

Fluorescence is common in both coral adult and larval stages, and is produced by fluorescent proteins that absorb higher energy light and emit lower energy light. This study investigated the changes of coral fluorescence in different life history stages and the effects of parental light environment on larval fluorescence, larval endosymbiotic dinoflagellate abundance, larval size and settlement in the brooding coral *Seriatopora hystrix*. Data showed that coral fluorescence changed during development from green in larvae to cyan in adult colonies. In larvae, two green fluorescent proteins (GFPs) co-occur where the peak emission of one GFP overlaps with the peak excitation of the second GFP allowing the potential for energy transfer. Coral larvae showed great variation in GFP fluorescence, dinoflagellate abundance, and size. There was no obvious relationship between green fluorescence intensity and dinoflagellate abundance, green fluorescence intensity and larval size, or dinoflagellate abundance and larval size. Larvae of parents from high and low light treatments showed similar green fluorescence intensity, yet small but significant differences in size, dinoflagellate abundance, and settlement. The large variation in larval physiology combined with subtle effects of parental environment on larval characteristics seem to indicate that even though adult corals produce larvae with a wide range of physiological capacities, these larvae can still show small preferences for settling in similar habitats as their parents. These data highlight the importance of environmental conditions at the onset of life history and parent colony effects on coral larvae.

## Introduction

Coral reefs are now threatened on a global scale due to anthropogenic climate change as well as local stressors [1], [2]. Scleractinian corals create the foundation of coral reefs. Therefore, the future of coral reefs, one of the most productive and diverse ecosystems on our planet, is dependent on the reproductive success of scleractinian corals. Most corals are broadcast-spawners, in which eggs are fertilized externally in the water column and larvae may be pelagic for long periods of time [3]. However, some common scleractinian corals are brooders [4], in which eggs are fertilized internally and larvae are capable of settling quickly, although larvae still have the potential to spend significant amounts of time in the water column and can remain competent for >100 d [5], [6]. In both cases, the physiology and ecology of the larva, and in particular its ability to settle in a favorable location, are ultimately essential in determining the fitness of an adult coral. The transition from pelagic and mobile larva to benthic and sessile adult is a critical life history change.

As coral reefs experience new pressures due to rapid changes in the environment, the phenotypic diversity of larvae is vital to provide the variation and range of tolerances necessary for natural selection. Both genetic and environmental variation may play a role in equipping larvae with a diversified toolkit for physiological adaptation. However, genetic variation is heritable while environmental effects are not. An example of genetic variation in coral larvae includes their response to elevated temperature [7]. Alternatively parental effects, which are the influences of both the parental environment and phenotype on the offspring phenotype, are fundamental to biological systems and can shape offspring development, behavior, and fitness [8], [9]. While parental effects are well studied in plants, insects, and terrestrial vertebrates [8], they are poorly considered in marine environments [9] and in particular corals [10]. Parental light environment has been shown both to influence larval survivorship in broadcast-spawners [11] and to not influence larval survivorship in the brooder *Pocillopora damicornis* [12]. However, both studies showed that there were higher concentrations of photoprotective compounds in larvae from parents of higher light environments [11], [12].

Because coral larvae and recruits (newly settled corals) are small and difficult to observe in the field, their autofluorescence has often been used as a tool to study them [13]–[15]. Corals have long been known to be fluorescent [16]–[18], which results from a family of fluorescent proteins that absorb higher-energy light and subsequently emit lower-energy light [19]–[21]. A whole color spectrum of fluorescent proteins has been identified in corals, but the green fluorescent protein (GFP) is most common and thought to be the ancestral protein [19], [22]. Fluorescence has been briefly noted for a variety of coral eggs [23]–[25], embryos in *Montastrea cavernosa* [24], coral larvae in *Stylophora pistillata* [26], first polyps in *Acropora millepora* [27], and in many families and genera of recent recruits [15]. In these cases, the coral fluorescence expressed during coral early life history stages appeared similar to the fluorescence expressed by adults. Consequently, any changes coinciding with developmental stages would then be considered an ontogenetic shift. There has been only one previous study carefully examining coral larvae fluorescence [28]. This study showed that larvae and adults of the broadcast-spawner *Acropora millepora* can express both red and green fluorescence with surprisingly no correlation between the parent and larval dominant color of fluorescence [28]. Furthermore, this study showed that larvae with more red fluorescence had reduced success of settlement and that heat stress reduced red fluorescence. These data suggest that coral fluorescence may reflect larvae ability to settle and to withstand stress, both of which are crucial for a larva to be successful [28].

The *in vivo* function of fluorescent proteins in corals remains unknown and controversial despite the widespread use of fluorescent proteins in cellular biology [29]. Originally, fluorescent proteins were proposed to have a photoprotective function [17], [30]. However, this hypothesis has been weakened by a lack of correlation between fluorescent protein abundance and depth of corals distribution [31], [32]. Another early hypothesis, photosynthesis enhancement [18], has been questioned because of the inefficient transfer of energy to dinoflagellates [32], [33]. Lack of clear evidence has led to alternative hypotheses including camouflage [34], antioxidant activity [35], [36], regulation of symbiotic dinoflagellates [37], [38], and being part of an innate immune response [39]. Recently, there has been increasing evidence that fluorescent proteins in corals are strongly influenced by light level and wavelength [27], [38], [40], [41]. During heat stress, a decrease in fluorescent protein transcripts were found in both larvae [42] and adults [43], [44], which may provide evidence for fluorescence to indicate stress and thus be used as a marker of deleterious physiological conditions. The close association between coral fluorescence and the abiotic factors of the surrounding environment suggest that fluorescent proteins play an important role in how corals interact with their habitat.

Our study investigated the fluorescence from different life history stages in the brooding coral *Seriatopora hystrix* and assessed the effects of parental light environment on coral larvae fluorescence, dinoflagellate abundance, size and settlement behavior. This study also examined the relationships between these characteristics and the variability of larvae found within an individual parent colony. Understanding the patterns of fluorescence during different life history stages may give insight into the functions of fluorescent proteins in corals, while also providing a better ecological understanding of the effect of light on the ontogeny of corals.

## Materials and Methods

### Sample Collection and Aquarium Design

*Seriatopora hystrix* is a common Indo-Pacific shallow-water coral with many color and branching morphologies, which may actually represent many cryptic species [45], [46]. Therefore, the brown color morph (under white light) of *S. hystrix* was used exclusively in this study to prevent different physiologies due to different color morphs [47]. Adult colonies of *S. hystrix* (*N* = 16, 14–22 cm in diameter) were collected from depths of 4–7 m Nanwan Bay in Kenting, Taiwan (21° 56′ 29″ N, 120° 44′ 70″ E) without prior knowledge of their fluorescence. Corals were collected under Kenting National Park permit number 0972903180. The colonies were collected about 8–10 m apart to avoid colonies of the same genotype. The Kenting coastal area has three river drainages and receives heavy rainfall in summer creating an ocean environment with high amounts of nutrients and suspended solids [48]. In Taiwan, *S. hystrix* is a hermaphroditic brooder producing larvae monthly throughout the year and generally larvae peak release is around the first quarter to the full moon of the lunar cycle [49]. Corals were collected six days after the full moon (13 July 2009) because brooding corals are likely to be fertilized two weeks or less prior to larvae release ([50]; pers. obs.) and peak release of larvae was predicted to be ∼15 d later.

At the National Museum of Marine Biology and Aquarium, adult corals were immediately placed in individual 10 L aquaria with flow through filtered seawater (∼6 mL sec^−l^) and an air bubble, and all parasitic snails were removed. The corals were maintained in an outdoor area (with transparent plastic ceiling) with natural photoperiod and at ambient reef temperatures (26–29°C in the aquarium; mean monthly seawater temperature in the field was 28°C during the time period of the experiment). The light in the tanks could reach 1,200 µmol quanta m^−2^ s^−1^ at the peak of the day with clear skies, which is consistent with the maximum light intensities reached at the collection site and depth. To test the effect of the parental environment on larval characteristics, adult coral colonies were maintained under two light environments (*N* = 8 per treatment). There was no further manipulation (as described above) for the high light treatment. The low light treatment was created using a neutral density shade cloth to reduce 85% of the sunlight; thus, light levels could reach 180 µmol quanta m^−2^ s^−1^ at peak day with clear skies. Corals were collected from the field and immediately placed into their respective light treatments. The outflow from each aquarium flowed to an individual larval collection cup [51]. The larvae were released pre-dawn [51] and the cups were collected ∼0800 hrs and examined for larvae. In this study, corals were in treatments for 13–17 d before larvae were collected ensuring that most of the larval development occurred while parent colonies were in different light treatments. Larvae used in this study were collected from 26–30 July 2009 (≥3 larvae per adult colony per day).

### Spectral Properties of Coral Fluorescence

The spectral characteristics of the coral adult and larval fluorescence were determined with a fluorescence spectrophotometer (F-2500, Hitachi, Tokyo, Japan) and spectrograph (Echelle SE200 Digital Spectrograph, Catalina Scientific, Tucson, AZ, USA). For the fluorescence spectrophotometer measurements, the coral tissue from a branch of the adult colony was removed with an artist’s airbrush and filtered seawater (*N* = 2 and confirmed with data from spectrograph where *N* = 16). Larvae from the same parent colony were pooled to obtain fluorescence measurements; only a subset of adults produced enough larvae to make measurements. Pooled intact live larvae in filtered seawater were measured (from 5 adult colonies). Excitation and emission spectra were normalized to the highest peak in the spectrum. Additionally, the emission spectra of all adult colonies were measured using a low-light digital spectrograph with a fiber optic probe placed about 2 mm from the live coral and excited with a variety of light wavelengths (EXFO X-Cite 120 W mercury lamp, Ontario, Canada), although cyan light (436±20 nm) systematically produced the best data and those results are presented in this study (*N* = 8 adults per treatment). A few adult colonies (*N* = 3) were visually checked for red fluorescence using green light flashlight source and red barrier filter glasses (Nightsea, Bedford, MA, USA), but no red fluorescent protein fluorescence was observed.

### Epifluorescence Microscopy and Image Analyses

Adults, recruits and larvae were imaged using an epifluorescence stereoscope (Nikon SMZ1500, Melville, NY, USA, with EXFO X-Cite 120 W mercury lamp) coupled to a color digital camera (Retiga 2000R, QImaging, Surrey, Canada). Each sample was observed with white light and three filter cube sets, DAPI (excitation 390±22 nm, emission 460±50 nm), cyan (excitation 436±20 nm, emission 480±40 nm), and blue (excitation 470±90 nm, longpass emission ≥500 nm). No fluorescence was observed in any sample with the DAPI filter cube and therefore fluorescent images were obtained only from cyan and blue filter cubes.

All images were processed in ImageJ software (National Institute of Health software, Bethesda, MD, USA) and only larvae in the lateral view were used for analyses (*N* = 91 from 8 adults for high light treatment, *N* = 61 from 7 adults for low light treatment). To determine the GFP fluorescence of larvae, the green channel image obtained with the blue filter cube set (exposure time 48.8 ms) was used to trace the outline of the larva and the average pixel intensity within that region was measured. The average background was also obtained for each image and subtracted from the average pixel intensity of each larva. Pixel resolution of fluorescence intensity was 8-bit, scaling from 0–255.

Images collected with white light illumination were used to determine the surface area of endosymbiotic dinoflagellates in each larva as a proxy for dinoflagellate abundance (Figure S1A). The dinoflagellate percent surface area of each larva was determined by tracing the edge of the larva and individually thresholding the image so that the surface area of the larva with dinoflagellates was selected. The blue channel 8-bit image was used for thresholding because it showed the most distinct separation amongst individual dinoflagellates (Figure S1B). The threshold was set to cover the area of the larva that contained dinoflagellates (Figure S1C). The measurement obtained was then the surface area of the dinoflagellate area relative to the whole surface area of the larva.

White light images were also used to determine the length, width, and 2-dimensional area of each larva. The length of the larva was measured as the longest distance from the oral to aboral end and the width of the larva was measured at the widest part of the larva orthogonal to the length. The area of a larva was calculated as the area of an ellipse A = π*ab*, where *a* is ½ length and *b* is ½ width.

### Settlement Behavior Experiment

Larvae settlement behavior experiments were performed in pre-soaked polystyrene 6- well culture plates. Half of each well (35 mm in diameter) was exposed to light and half was covered in black tape that was impenetrable to light. The culture plates were placed under fluorescent light bulbs with a photosynthetically active radiation (PAR) irradiance of 230 µmol quanta m^−2^ s^−1^ on a 12∶12 h light:dark photoperiod and in water baths (27°C) to maintain constant temperature. Ten larvae from a single parent colony were placed in a well with 13 mL of filtered seawater. The high light treatment had 391 larvae in 39 wells with larvae from high light adults (representing 7 adult colonies) and the low light treatment had 328 larvae in 33 wells (representing 6 adult colonies). After 24 hrs, the location of larval settlement was characterized as the following: settled in high light (exposed half), settled in low light (covered half), settled along the light/dark border (∼2 mm on either side of the border), unattached (underwent metamorphosis but not attached to the substrate), and swimming (no metamorphosis). The larvae that settled in high and low light habitats were of primary focus in this experiment to determine whether the larvae had settlement preferences for lighter or darker habitats based on their parental history.

### Data Analysis

All statistical analyses were conducted using JMP version 8.0 (SAS Institute, Inc., Cary, NC, USA). Data sets were tested for assumptions of normality and homoscedasticity, and data were transformed accordingly prior to analyses. T-tests were used to compare the adult fluorescence emission peaks as determined by the spectrophotometer and spectrograph (see above). Nested analysis of variance (nested ANOVA) tests were used to test the effect of the parental environment and parent colony on GFP fluorescence, dinoflagellate abundance, and larval size. For the nested ANOVAs, data from two low light colonies were excluded because the sample size was only 1 larva each. The percentage of variation from the parent colony and parent treatment was determined by calculating the ratio between the sum of squares associated with the factor and the total sum of squares (all possible factors of variation+residual; [52]). Simple correlation analysis was used to test the relationship between GFP and dinoflagellate abundance, larvae area and dinoflagellate abundance, and GFP fluorescence and larvae size for both low light and high light larvae. The results of the settlement experiment were presented as the percentage settled in high and low light by wells (with ∼10 larvae per well). Pearson’s chi-square test of the contingency table on the raw larvae data (non-percentage) was used to test the effect of the parental environment on the settlement experiment. A t-test was also used to evaluate whether there was a difference in the percentage of larvae swimming between high and low adults at the end of the settlement experiment. Averages represent arithmetic means ± standard errors. Statistical differences were significant at the α = 0.05 level.

## Results

**Figure 1:**
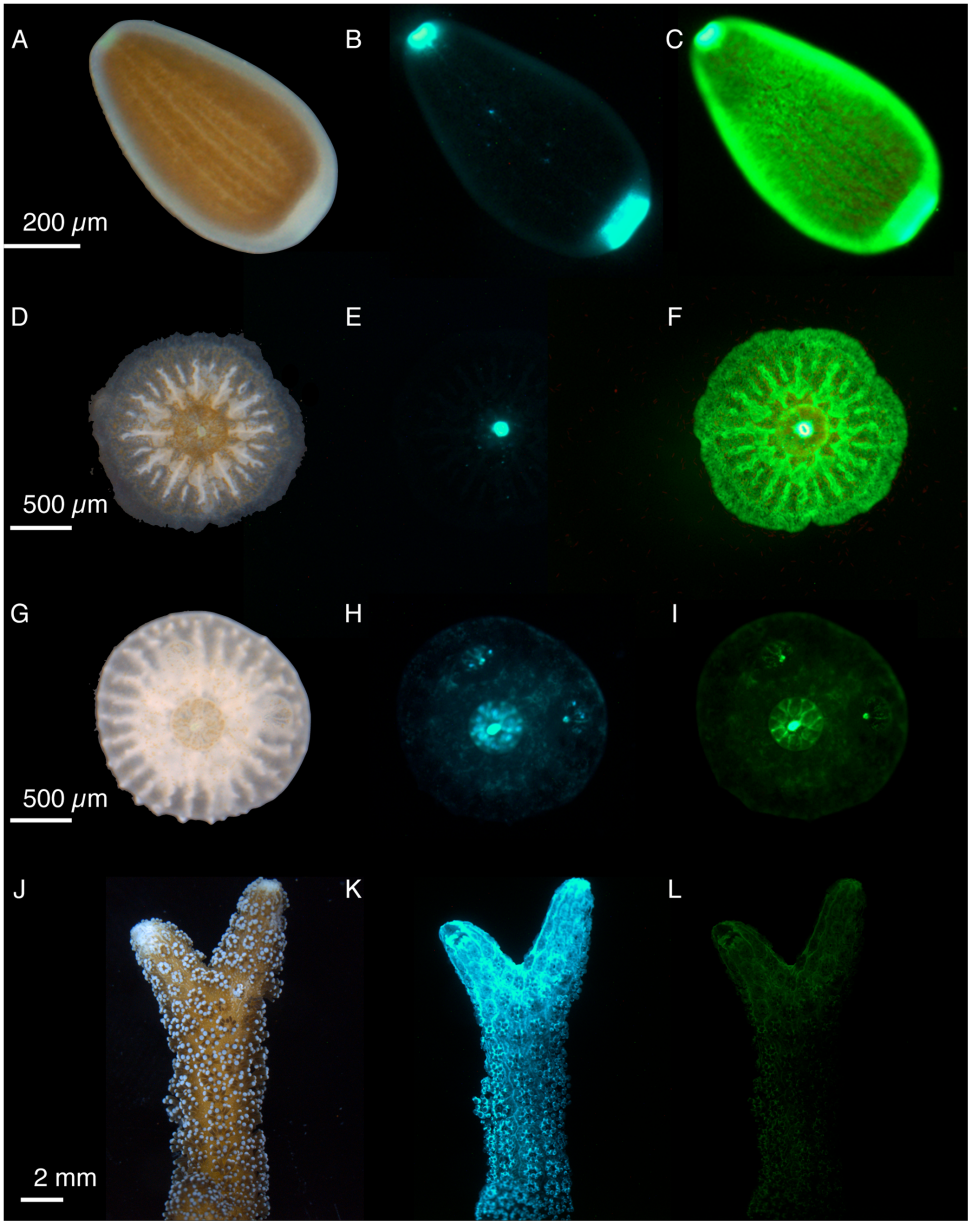
Life history changes in coral fluorescence in *Seriatopora hystrix.* Images representing life history stages including (**A**–**C**) larva, (**D**–**F**) 1 d recruit, (**G**–**I**) 14 d recruit, and (**J**–**L**) adult under (**A**, **D**, **G**, **J**) white light, (**B**, **E**, **H**, **K**) cyan light (excitation 436±20 nm and interference filter 480±40); and (**C**, **F**, **I**, **L**) blue light (excitation 470±40 nm and longpass emission filter ≥500 nm). Cyan fluorescence images (**B**, **E**, **H**, **K)** exposure times were 700.7 ms and green fluorescence images exposure times were (**C, L**) 48.8 ms and (**F, I**) 137.7 ms.

**Figure 2:**
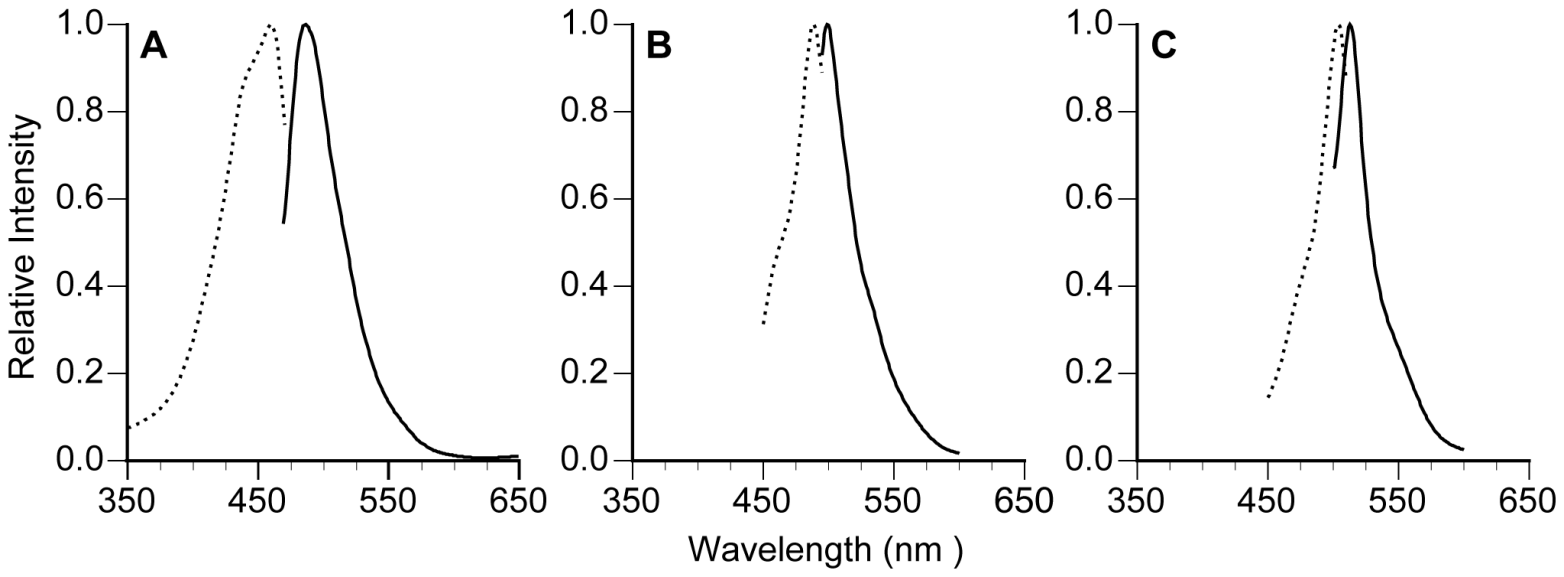
Spectral characteristics of fluorescence in *Seriatopora hystrix* (A) adults and (B–C) larvae. Dotted line represents excitation spectra; solid line represents emission spectra.

*Seriatopora hystrix* showed distinct fluorescence patterns at different life history stages, most likely a result of dissimilar FP expression (Figure 1, Figure 2). The adult colonies of *S. hystrix* display cyan fluorescence throughout the coenosarc and the polyp. In contrast, the larvae expressed green fluorescence throughout the whole larvae, with higher concentrations at the oral and aboral ends as compared to the middle of the larva, being 5–27× greater in the oral end and 3–23× greater in the aboral end (*N* = 4 per treatment; note that the oral end was often underestimated because of pixel saturation). The adult colonies of *S. hystrix* displayed a single cyan fluorescent protein (CFP) with an excitation peak of 459.8±0.3 nm and an emission peak of 486.5±0.5 nm (Figure 2A; spectrophotometry data from extracted samples). These results are similar to the emission peak measured with the spectrograph on all the live adult colonies of 484.3±0.4 nm with no significant difference between the two methodologies (t_16_ = 2.0, *P* = 0.07). Because the CFP emission peak is broad and extends well past 500 nm, the CFP is also responsible for the apparent green fluorescence in adults (Figure 1L). In contrast the larvae expressed two green fluorescent proteins (GFPs) with excitation peaks at 489.5±0.5 nm and 504±0 nm and emission peaks 499.3±0.3 nm and 512.7±0.3 nm, respectively (Figure 2B–C). The two GFPs appear to be located in spatially distinct regions with the higher energy GFP restricted to the poles (Figure 1B,C). Larvae were collected from high and low light parents and the two types of GFP were observed in larvae from both parental treatments. The recruits also expressed GFP (Figure 1F), but a full spectral characterization was not possible because they were attached to the substrate. It was clear however, that *S. hystrix* displayed an ontogenetic development pattern of coral fluorescence.

**Figure 3:**
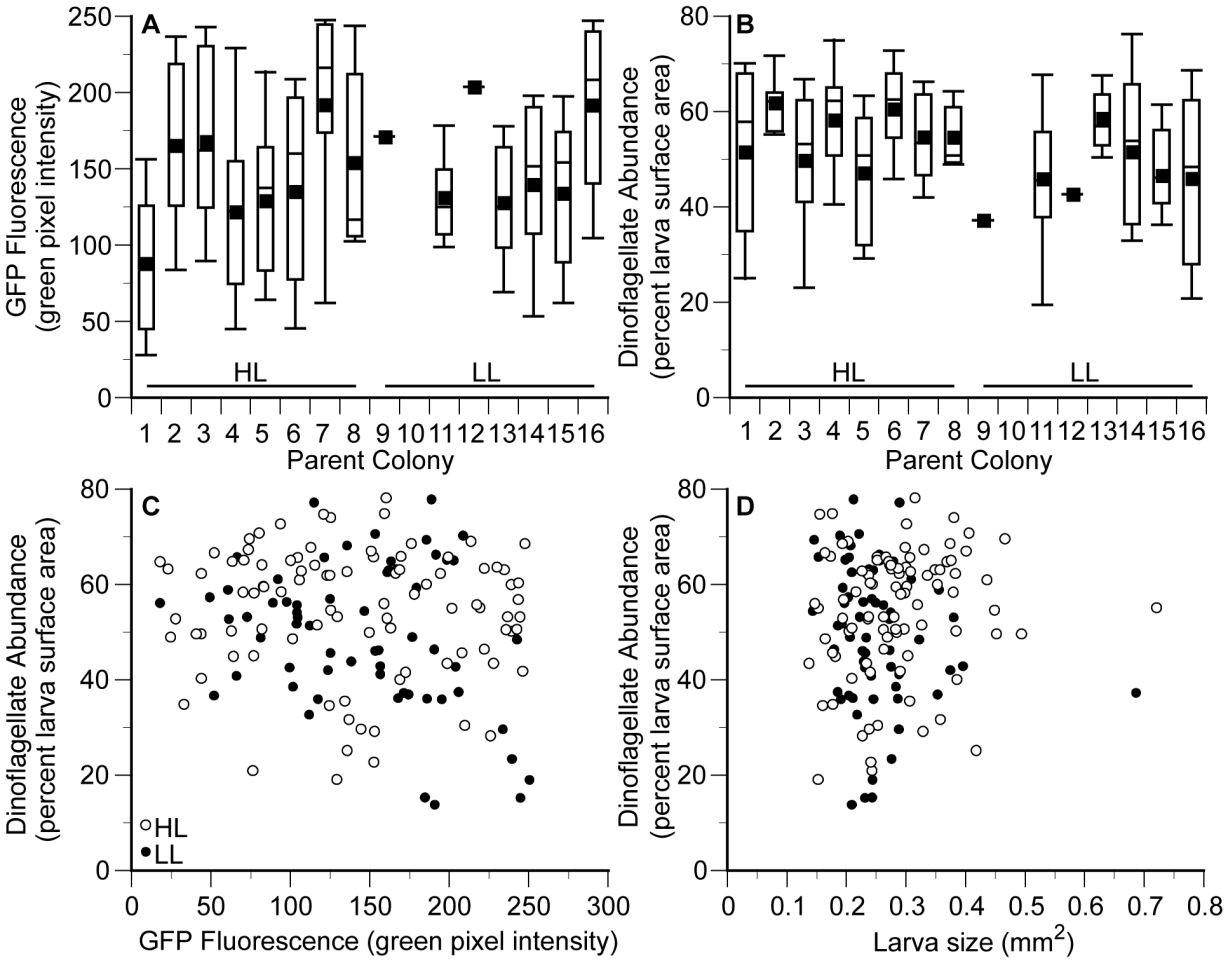
*Seriatopora hystrix* larval characteristics. Box plot of green fluorescent protein (GFP) fluorescence (**A**) and dinoflagellate abundance (**B**) in larvae produced by 16 parent colonies (high light (HL) colonies 1–8 *N* = 14, 13, 9, 15, 14, 9, 14, 3, respectively; low light (LL) colonies 9–16 *N* = 1, 0, 7, 1, 9, 16, 18, 9 respectively). Errors bars represent deciles (10^th^ and 90^th^ percentiles), white boxes represent quartiles (25^th^, 50^th^, and 75^th^ percentiles), small black boxes represent arithmetic means. (**C**) and (**D**) examine the relationships between larval characteristics. Each point represents an individual larva and open circles represent larvae from high light parents and dark circles represent larvae from low light parents. (**C**) Larval fluorescence is not related to dinoflagellate abundance in larvae from high light parents (F_1,89_ = 0.3, *P* = 0.60, R^2^ = 0.003), but there is a weak relationship between larval fluorescence and dinoflagellate abundance in larvae from low light parents (F_1,59_ = 5.2, *P*<0.05, R^2^ = 0.08). (**D**) Dinoflagellate abundance is not related to larval size (high light larvae: F_1,89_ = 1.9, *P* = 0.17, R^2^ = 0.02, low light larvae: F_1,59_ = 2.0, *P* = 0.17, R^2^ = 0.03).

**Figure 4:**
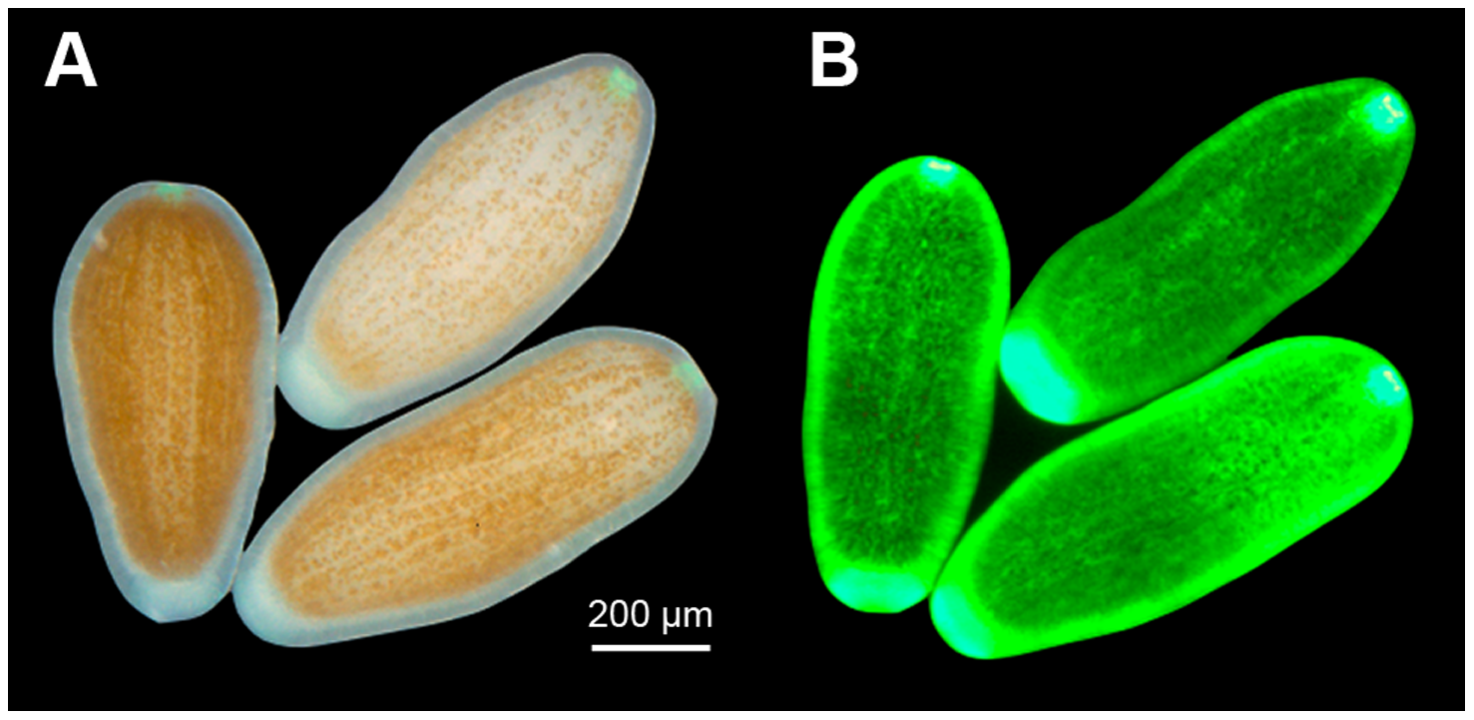
*Seriatopora hystrix* coral larvae showing variable dinoflagellate density but similar green fluorescent protein (GFP) fluorescence. Larvae from one parent colony under white (**A**) and blue light (**B**) showed similar GFP fluorescence (left to right: 153, 185, 138 green pixel intensity) regardless of dinoflagellate abundance (left to right: 71, 15, 44% larva surface area).

In general, larvae GFP fluorescence, dinoflagellate abundance and size were incredibly variable. GFP fluorescence in larvae from high and low light adults ranged 13 and 14-fold respectively, and larvae from the same individual parent colony ranged nearly 6-fold (Figure 3A). The parent treatment did not have a significant effect on larval GFP fluorescence (F_1,137_ = 0.33, *P* = 0.57), but parent colony did have a significant effect (F_11,137_ = 2.85, *P*<0.01). However, only 18.6% of the variation was accounted for by the parent colony and leaving large amount of the variation unaccounted for. Dinoflagellate abundance in larvae from high and low light adults ranged 4 and 5.5-fold respectively, and also varied largely within a single parent colony (Figure 3B, Figure 4A). The parent treatment did have a significant effect on dinoflagellate abundance (F_1,137_ = 4.06, *P*<0.05), but it only accounted for 2.6% of the variation. Additionally, the parent colony did not have significant effect on dinoflagellate abundance (F_11,137_ = 1.63 *P* = 0.10). Size in larvae from high and low light adults ranged 5-fold in both treatments and larvae were significantly different between parent treatment (F_1,137_ = 5.06, *P*<0.05) and parent colony (F_11,137_ = 2.68, *P*<0.01). However, only 2.9% of the variation could be attributed to the parent treatment and 17.2% of the variation could be attributed to the parent colony.

There was a significant relationship observed between larvae GFP fluorescence and dinoflagellate abundance in larvae from low light adults (F_1,59_ = 5.2, *P*<0.05, R^2^ = 0.08); however, the relationship was not significant in larvae from high light adults (F_1,89_ = 0.3, *P* = 0.60, R^2^ = 0.003) (Figure 3C). Three larvae released by an individual parent colony on the same night exemplify the amount of variation in dinoflagellate abundance and the weak correlation with GFP fluorescence (Figure 4). In larvae from both high and low light adults, larval size and dinoflagellate abundance were not significantly correlated based on a least-squares linear regression (Figure 3D; respectively, F_1,89_ = 1.9, *P* = 0.17, R^2^ = 0.02, F_1,59_ = 2.0, *P* = 0.17, R^2^ = 0.03). Furthermore, in larvae from both high and low light adults there was no significant relationship between GFP fluorescence and larval size based on a least-squares linear regression (respectively, F_1,89_ = 1.89, *P* = 0.17, R^2^ = 0.02, F_1,59_ = 0.1, *P* = 0.74, R^2^ = 0.002).

**Figure 5:**
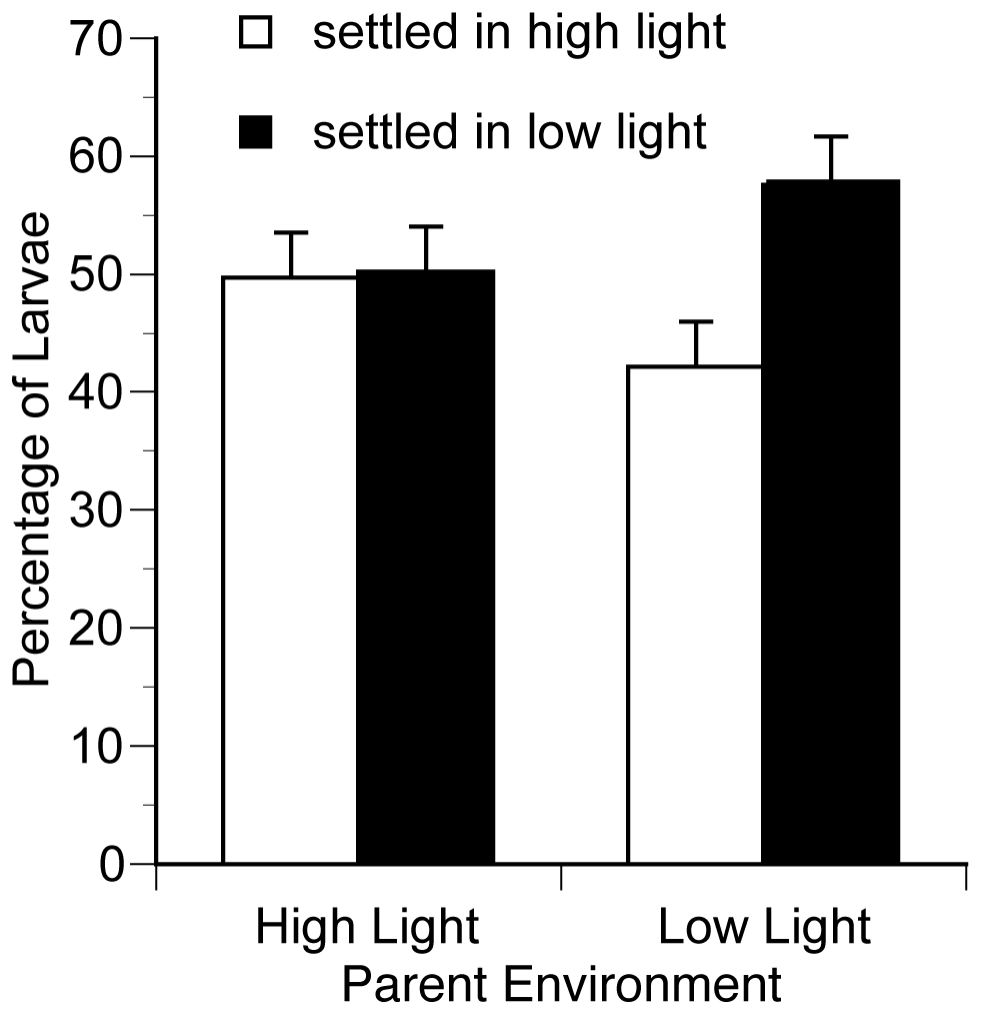
*Seriatopora hystrix* coral larvae settlement preference experiment. Larvae, from parent colonies acclimated to high (*N* = 39 wells) or low light (*N* = 33 wells) environments, could settle in high light or low light conditions. Pearson’s chi square test indicated significantly different larvae settlement frequency distributions depending on parent environment.

Considering the larvae that settled in either low or high light environments, the larvae settlement experiment showed small but significant differences between the larvae from parents in different environments (Figure 5). The frequencies of larvae settlement in low and high light habitats were significantly different between the parental environments (χ^2^ = 4.1, *N* = 554, *P*<0.05), and there was a greater probability for larvae to settle in low light habitats if the parent was from a low light environment. The larvae brooded under low light conditions settled more under low light (57.9±5.0%) than under high light (42.1±5.0%), while the larvae brooded under high light conditions showed no preference between settling in high (49.7±4.0%) and low light (50.3±4.0%) environments. Considering all the larvae, there were a few larvae that settled in the border, were unattached or still swimming from both the high (respectively 15%, 0.3%, and 7.4%) and low light parents (respectively 7.9%, 2.1%, and 13.7%). Although larvae from high light parents had a higher propensity for settlement compared to larvae from low light parents, the percentage of swimming larvae between the two treatments was not statistically different at the end of the settlement experiment (t_60_ = 1.26, *P* = 0.21).

## Discussion

*Seriatopora hystrix* displayed changes in fluorescence with life history stage. To our knowledge, this study is the first to show an ontogenetic shift in coral fluorescence expression. Larvae displayed bright green fluorescence and contained two GFPs, whereas adult corals expressed cyan fluorescence and accordingly had a single CFP. Previous literature provides evidence for similar fluorescence in coral eggs and adults in *Montipora capitata* [25], embryos and adults in *Montastrea cavernosa* [24], and larvae and adults in *Acropora millepora* [28]. Analogous to *S. hystrix*, the cephalochordate amphioxus has 16 types of GFPs and expresses different GFPs during different life history stages [53]. Additionally, the spatial pattern and location of expressed GFP differs in amphioxus larvae and adults [54].

The fluorescence of adult corals is blue shifted as compared to the fluorescence expressed by the larvae (CFP: 460 nm excitation vs. GFPs: 490 nm and 504 nm excitation). Because blue light travels deeper, sessile adult corals may experience light regimes that are blue shifted compared to the swimming larvae. While the FPs of the larvae and adults seem to match their respective light environments, the apparent lack of functional CFP in the larvae provides evidence against a photoprotective role in FPs. Indeed, larvae in the water column would experience higher levels of blue light than the adults on the benthos, and therefore, need greater photoprotection in that region of the spectrum. Instead these data may favor of a possible biochemical function for FPs. The emission peak of the first GFP of the larvae (499 nm) coincides with the excitation peak of the second GFP (504 nm). The overlap of the emission and excitation of the two GFPs would allow for the potential of a cascade of energy transfer between the two GFPs [30]. The transfer of energy between GFPs would however depend on tight proximity, and further research to address the natural occurrence of such energy transfer in cascade should consider fine scale spatial and spectral mapping of GFPs. Furthermore, the possible biochemical, ecological and/or metabolic function/s associated with such transformation of photons at the molecular and proteomic level still require additional investigation.

*S. hystrix* larvae showed large ranges of coral fluorescence (≥13-fold), dinoflagellate abundance (≥4-fold) and size (≥5-fold), even among larvae produced by the same parent colony or from the same parent light environment. The large variation of dinoflagellate abundance in larvae in this study is similar to what has been observed in brooding corals [55], [56] including a >4-fold range in *S. hystrix* [56]. While larvae with less dinoflagellates initially have lower rates of photosynthesis, the number of dinoflagellates increases quickly so that there is no difference in dinoflagellate abundance between larvae with originally low or high abundance after 3 weeks [55], indicating dinoflagellate abundance upon larval release may not be a critical factor for larval fitness.

There are several possible explanations for high variability of larval characteristics in this species. Within an adult branching coral, there are many different light microhabitats created by branches and light can differ by 50-fold, which causes differences in productivity [57]. Moreover, the age of the branch can also have large consequences on the density and size of polyps, dinoflagellate abundances and photosynthetic capacity [57]. Healthy adult corals living at the same depths can also show a large range of dinoflagellate pigment concentration (1.5–10-fold) and dinoflagellate abundance (1.3–8.8-fold) [58]. Additionally, there can be high genetic variability in larvae released from an individual coral colony because fertilization can result from multiple sires as well as selfing in *S. hystrix* [59]. These environmental and genetic differences within an individual parent colony may contribute to the observed variability in the coral larvae.

Because of the large amount of variation in coral larvae, it was not surprising that there were relatively few differences in larvae from high and low light parent colonies. Nevertheless, there were small but significant effects of parent treatment on dinoflagellate abundance (3% of the variation) and size (3% of the variation). If adult corals were maintained in different treatments for more time there may have been larger differences between the larvae from different parental treatments; however, this was not possible in this experiment because adult corals were collected shortly after fertilization and collecting them earlier would have caused much lower amounts of larvae to be produced, based on past experience (T.Y Fan, unpubl. data). However, it is probable that the larvae developed mostly if not entirely while the parents were in their respective light environments. Interestingly, the effect of the parent colony was larger than the effect of the parental treatment, thus emphasizing the importance of the genetic contribution compared to the environmental factor. Noticeably, parent colony explained 17% of the variation in larval size, which is nearly 6× higher than the parent treatment contribution. In GFP fluorescence, there was no significant parent treatment effect, but parent colony contribution explained 19% of the variation, which was similar to what was reported *in Acropora millepora,* for which the emergence of a specific color of fluorescent protein was proposed to be a predictor of settlement success [28].

Given all the larvae variation highlighted in this study, the small but significant differences between the larvae from different light treatments appeared important. The larvae from high light parents were slightly larger and had slightly higher dinoflagellate abundance than the larvae from low light parents. One advantage to size is that larger larvae have higher survivorship rates than smaller larvae [56]. Because the variation in larval size increases as there are more larvae [56] and larvae frequently change shape [26], it places greater importance on the measured differences in size between larvae from different parental treatments. Although the larvae from high light parents were equally likely to settle in high and low light habitats, they were more likely to settle in higher light environments than the larvae from low light parents. Assuming equal rates of survivorship, the larvae that settle in higher light environments may grow more quickly. It is also possible the differences in larvae settlement would have been more pronounced had higher light intensities been used instead, yet our study targeted the realistic representation of field conditions.

Previous studies have shown that there was no effect of parents from different photosynthetically active radiation and ultraviolet radiation light environments on larvae settlement or mortality [12], [60]. However, the light intensity and spectral quality of light can have important consequences for larvae settlement depending on the parent depth distribution [61]. In Southern Taiwan, *S. hystrix* is more typically found at depths of 4–12 m, while *Pocillopora damicornis* is more common at shallower depths (1–5 m) (T.Y Fan, unpubl. data), yet it is unknown whether settlement preferences or differential post-settlement mortality is the cause of such distribution patterns. Large larval variation and the subtle differences of larvae from different parental environments found in this study suggest that the adult coral colonies are producing larvae that have the physiological capacity to settle in a variety of habitats, but that larvae may have settlement preferences for environments similar to that of their parents.

## Supporting Information

Figure S1 **Different steps to quantify dinoflagellate surface area as a proxy for larva dinoflagellate density.** (**A**) Image of larva under white illumination, (**B**) the blue channel image, and (**C**) the blue channel image with outline the larva traced (in yellow) and the threshold adjusted to quantify the percentage of the dinoflagellate abundance, in this case 61% larva surface area.
(TIF)
