# Supplementary figures and images for: Life History Changes in Coral Fluorescence and the Effects of Light Intensity on Larval Physiology and Settlement in Seriatopora hystrix
Source: PLoS One. 2013 Mar 27;8(3):e59476. doi: 10.1371/journal.pone.0059476 (PMC3609816; doi:10.1371/journal.pone.0059476)

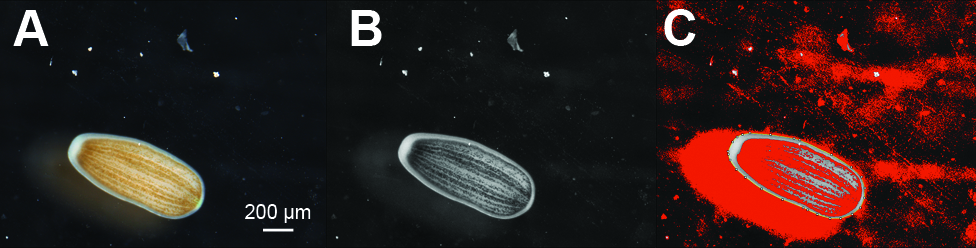

Supplement: Figure S1 — Different steps to quantify dinoflagellate surface area as a proxy for larva dinoflagellate density. (A) Image of larva under white illumination, (B) the blue channel image, and (C) the blue channel image with outline the larva traced (in yellow) and the threshold adjusted to quantify the percentage of the dinoflagellate abundance, in this case 61% larva surface area. (TIF) [file pone.0059476.s001.tif]
